# Divide and summarize: improve SLM text summarization

**DOI:** 10.3389/frai.2025.1604034

**Published:** 2025-08-01

**Authors:** Alexandre Bailly, Antoine Saubin, Gabriel Kocevar, Jonathan Bodin

**Affiliations:** Seenovate, Paris, France

**Keywords:** small language models, text summarization, Lost in the Middle, text generation, automatic evaluation

## Abstract

**Introduction:**

Text summarization is a longstanding challenge in natural language processing, with recent advancements driven by the adoption of Large Language Models (LLMs) and Small Language Models (SLMs). Despite these developments, issues such as the “Lost in the Middle” problem—where LLMs tend to overlook information in the middle of lengthy prompts—persist. Traditional summarization, often termed the “Stuff” method, processes an entire text in a single pass. In contrast, the “Map” method divides the text into segments, summarizes each independently, and then synthesizes these partial summaries into a final output, potentially mitigating the “Lost in the Middle” issue. This study investigates whether the Map method outperforms the Stuff method for texts that fit within the context window of SLMs and assesses its effectiveness in addressing the “Lost in the Middle” problem.

**Methods:**

We conducted a two-part investigation: first, a simulation study using generated texts, paired with an automated fact-retrieval evaluation to eliminate the need for human assessment; second, a practical study summarizing scientific papers.

**Results:**

Results from both studies demonstrate that the Map method produces summaries that are at least as accurate as those from the Stuff method. Notably, the Map method excels at retaining key facts from the beginning and middle of texts, unlike the Stuff method, suggesting its superiority for SLM-based summarization of smaller texts. Additionally, SLMs using the Map method achieved performance comparable to LLMs using the Stuff method, highlighting its practical utility.

**Discussion:**

Both theoretical and practical studies suggest that using Map method for summarization with SLM allowed to address the “Lost in the Middle” problem and outperform Stuff method.

## 1 Introduction

The creation of summaries has long been a challenge, but the use of Large Language Models (LLMs) to generate summaries has undergone a significant shift in recent years (Zhang et al., 2024). This change stems from the remarkable ability of LLMs to capture nuanced semantic relationships and contextual information across vast datasets (Brown, 2020). An experiment by Pu et al. (2023) compared summaries generated by humans and by LLMs through human evaluation, finding that LLM-generated summaries were preferred by participants.

However, LLMs have limited context size, meaning that the longest texts cannot be processed all at once, such as in Long Document Summarization tasks (Koh et al., 2022). Usually, documents are considered as long if they exceed the context length of classical Transformers models such as BERT (Vaswani et al., 2017; Devlin et al., 2018). To efficiently process documents without truncation, a novel approach, termed the “block method,” has emerged. It divides texts into smaller, manageable segments for individual summarization before synthesizing these partial summaries into a cohesive whole (Chang et al., 2023; Wu et al., 2021). This method provides a promising alternative to the traditional approach of processing the entire text in a single prompt, effectively mitigating context size limitations. This method, including the way in which the text must be segmented, has been thoroughly studied in works such as Moro et al. (2023) and Zhang et al. (2022).

Nowadays, the context size of LLMs has increased exponentially, enabling them to process long documents in one iteration. Despite the growing popularity of LLMs for text summarization, several challenges remain (Ghinassi et al., 2024). Among the most prominent are the “Lost in the Middle” problem (Liu et al., 2024) and the difficulty of evaluating generated summaries (Sai et al., 2022). The “Lost in the Middle” problem describes LLMs' tendency to overlook or underemphasize content in the middle of the prompt, resulting in incomplete or biased summaries. This issue is particularly critical for long texts that approach the LLM's context window limit. To the best of our knowledge, no studies have compared the benefits of the block method against the traditional method for shorter texts that fit within the context window of LLMs. We hypothesize that this segmented approach may mitigate the “Lost in the Middle” problem that fit within the prompt size. By processing smaller sections of text sequentially, the block method ensures that middle sections receive sufficient attention, resulting in more balanced and accurate summaries. In this article, the traditional global method and the block method will be referred to as “Stuff” and “Map,” respectively.

Despite their relatively limited number of parameters, Small Language Models (SLMs) have proven to be as effective as LLMs in certain contexts, such as content moderation (Ghinassi et al., 2024; Zhan et al., 2024). Their lightweight nature enables faster inference and lower computational overhead, making them well-suited for resource-constrained environments.

This study addresses two primary questions: (1) whether the Map method with a naive segmentation outperforms the Stuff method for texts that fit within the context window of SLMs, and (2) whether the Map method can effectively resolve the Lost in the Middle issue. To ensure broad accessibility and practical relevance, we focus on SLMs with fewer than 10 billion parameters, which are open-source and widely available. The SLMs are compared to a LLM to determine if the Map method enables them to bridge the performance gap between the two. To eliminate the need for human evaluation and to promote systemization, a simulation-based framework has been developed. This framework generates texts with a known number of facts and, for evaluation, quantifies the proportion of retained and omitted facts in summaries, offering an alternative to traditional metrics. This framework is applied to answer the two questions.

To validate the findings with real-world data, a comparative study was conducted using a corpus of scientific articles. The Stuff and Map methods were assessed based on their semantic similarity to the authors' abstracts. By addressing these questions and challenges, this work seeks to illuminate the trade-offs and benefits of different summarization approaches in the context of SLMs.

## 2 Materials and methods

### 2.1 Summarization methods

**Figure 1:**
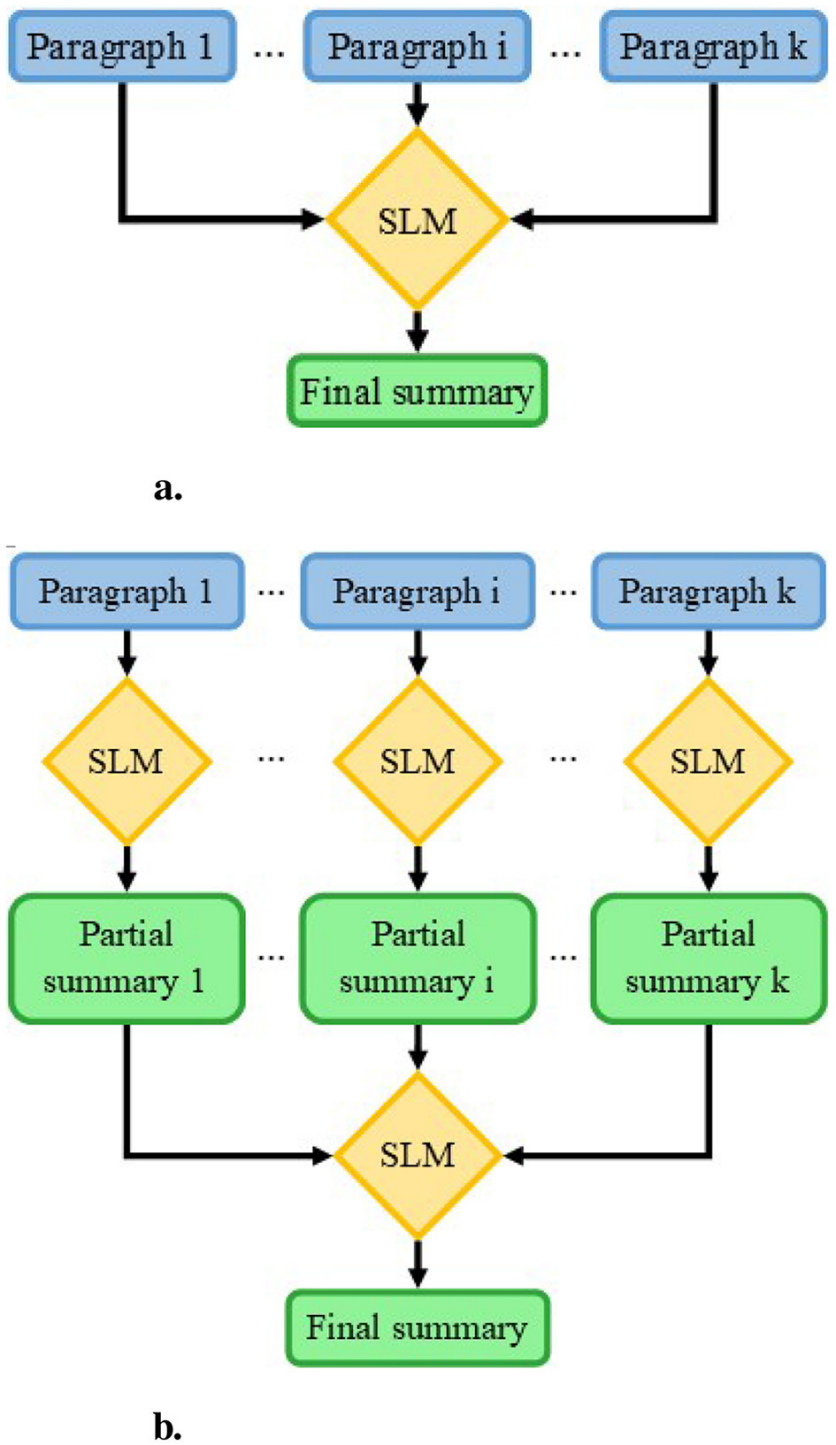
Schemas of summarization methods. **(a)** Stuff method: all parts of the text are provided to the SLM to obtain the final summary. **(b)** Map method: the text is first split into paragraphs which are independently summarized. The partial summaries are together summarized to obtain the final summary.

To evaluate the benefits of splitting text prior to summarization, two summarization methods using small language models (SLMs) were investigated, as illustrated in Figure 1. The first method, Stuff (Figure 1a), the text was summarized all at once, whereas in the second, Map (Figure 1b), portions of the whole text were summarized before summarizing the intermediate summaries.

#### 2.1.1 Stuff method

The Stuff method involves processing all the input text in a single pass. The complete text is provided to the SLM within the prompt,^1^ accompanied by a request to summarize it, as shown in Figure 1a. This approach is constrained by the initial input size; given the SLM's limited context length (around 8,000 tokens), the input cannot exceed this threshold.

#### 2.1.2 Map method

The Map method, initially introduced by Wu et al. (2021) and further explored by Chang et al. (2023), originally involves multiple summarization steps. In this study, the method has been simplified into two steps, as depicted in Figure 1b. First, the text is divided into multiple sub-texts, each consisting of a unique paragraph. These paragraphs are then individually submitted to the SLM^2^ with a request to summarize them, following the approach of the Stuff method. In the second step, all the sub-summaries are combined and provided to the SLM, along with an explanation that they represent parts of a larger text, and a request to produce a comprehensive summary of the original text. This segmentation overcomes context length limitations, enabling the processing of very long texts while potentially benefiting smaller texts as well.

#### 2.1.3 Studied SLMs

The two summarization methods outlined above were applied using several widely recognized SLMs from various companies, differing in size (measured by the number of parameters). The list of SLMs, along with their sizes and release dates, is provided in Table 1. The smallest SLM, Gemma2:2b, has 2.6 billion parameters, while the largest, Gemma2:9b, has 9.2 billion parameters. The earliest model, Openhermes:v2.5, was released in February 2023, and the most recent, Llama3.2, was released in September 2024.

**Table 1:** List of SLMs used in the studies with their respective sizes and release date.

**Model name**	**Size**	**Release date**
Gemma2:2b (Riviere et al., 2024)	2.6b	July 2024
Llama3.2 (Dubey et al., 2024)	3.2b	September 2024
Openhermes:v2.5 (Jiang et al., 2023)	7.0b	February 2023
Llama3.1 (Dubey et al., 2024)	8.0b	July 2024
Gemma2:9b (Riviere et al., 2024)	9.2b	June 2024

### 2.2 Simulation analysis

**Figure 2:**
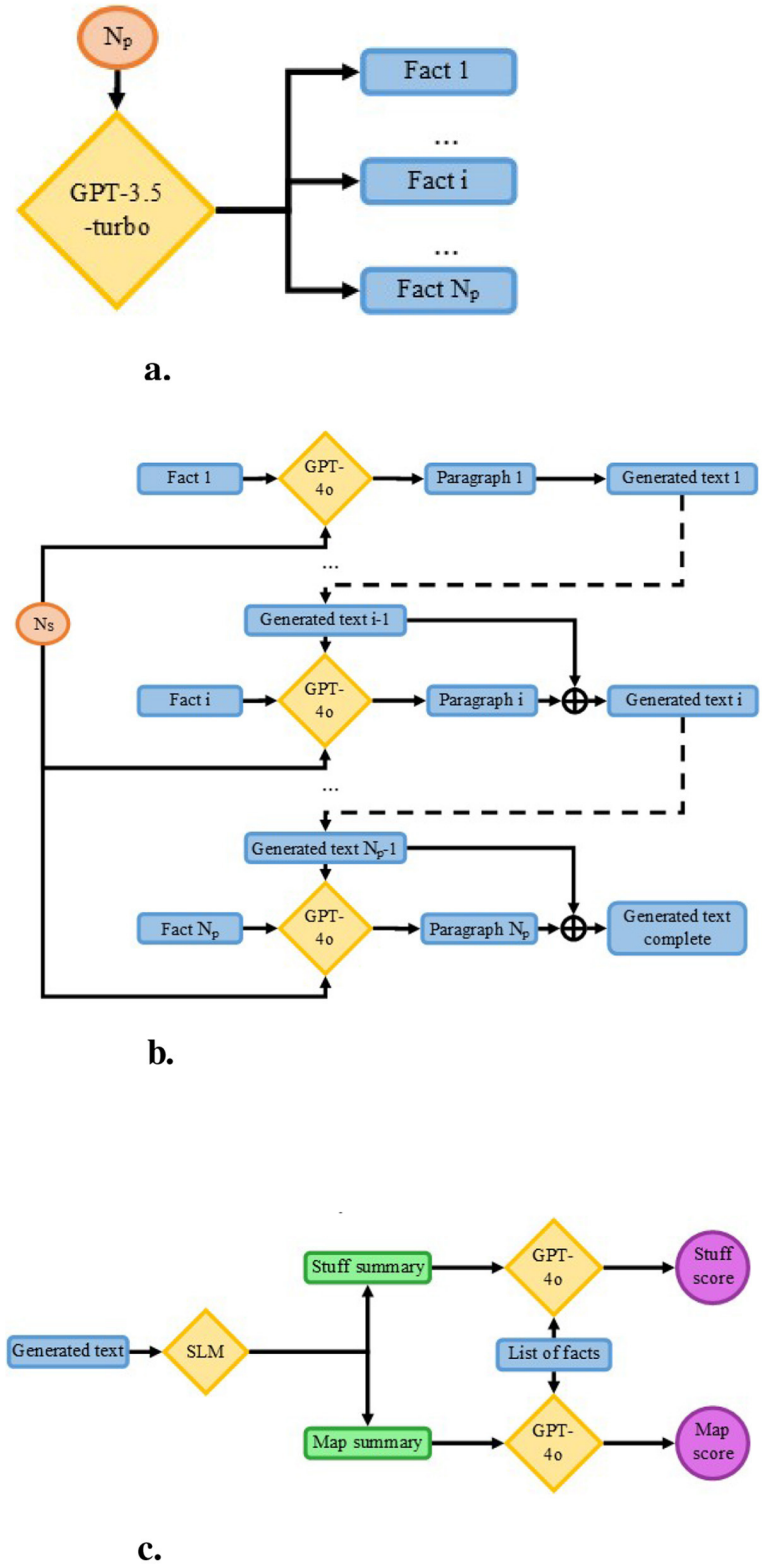
Simulation study pipeline, ⊕ represents the concatenate operator. **(a)** Creation of the list of facts from only a number facts targeted (*N*_*p*_). **(b)** Generation of text, paragraph by paragraph, each iteration taking as input the fact representing the main topic, the number of sentences targeted (*N*_*s*_) and the previously generated paragraphs to ensure continuity. **(c)** Summarization and evaluation process, each text was summarized using both the Stuff and the Map methods and were provided to GPT-4o with the list of facts to retrieve within the summaries.

In the initial phase, a study using simulated data was conducted to compare both methods on controlled texts with a fixed number of paragraphs and consistent paragraph sizes, as shown in Figure 2. The primary goal was to eliminate variability in text length within the study. To generate the simulated data, an original protocol was developed. First, a list of facts was created to define the topic of each paragraph. Next, a text was produced based on this list of facts and summarized using each method. Finally, each summary was assessed to compare the performance of the methods.

A minimum of 5 sentences per paragraph was required, as fewer sentences would be irrelevant and likely contain only the targeted fact. The maximum was set at 15 sentences, as 12 paragraphs with 15 sentences each approach the context limit of approximately 6,000 tokens for some SLMs. Furthermore, generating paragraphs longer than 15 sentences increases the risk of including facts that may be more significant than the targeted fact, potentially introducing bias into the analyses.

Additionally, using multiples of 3 for the number of paragraphs enabled us to easily divide the original texts and their corresponding facts into three parts (beginning, middle, and end) for positional analysis.

#### 2.2.1 Simulation plan

Since the length of input text may influence each summarization method, texts with varying numbers of paragraphs and paragraph sizes were examined. This was achieved by generating texts with 6, 9, or 12 paragraphs, each containing 5, 10, or 15 sentences. Only texts with uniform paragraph lengths were included, ensuring that each paragraph consisted of the same number of sentences. For each parameter combination (number of paragraphs × number of sentences), 30 texts were produced.

#### 2.2.2 Data generation

Several steps composed the text generation pipeline. Initially, lists of facts were produced using a LLM. These facts served as the main topic for each generated paragraph, with the number of facts matching the number of desired paragraphs. Subsequently, texts were created paragraph by paragraph, with the LLM instructed to adhere to a specified number of sentences per paragraph.

##### 2.2.2.1 List of facts generation

The initial step in data generation involved creating lists of facts, each serving as the main topic for a paragraph. Since texts were required to consist of 6, 9, or 12 paragraphs, the lists contained 6, 9, or 12 facts accordingly. For each paragraph count, 30 lists were produced, with the same lists applied to generate texts across all paragraph sizes.

Generation was performed using GPT-3.5-turbo-0125,^3^ as depicted in Figure 2a. Due to the model's generation length constraints, producing all lists simultaneously was not feasible, necessitating multiple calls to the model. However, repeated use of the same prompt yielded similar lists. To address this issue, the LLM was instructed to generate 15 lists per call, with only the last 10 retained. This process was repeated three times for each paragraph count, resulting in 30 lists per number of paragraphs.

During prompt engineering, a few-shot prompting technique was employed, incorporating two example generations for the LLM.

We manually reviewed all lists of facts to eliminate duplicates and ensure that facts within each list were distinct. When necessary, additional lists were generated until a suitable list was obtained and validated.

##### 2.2.2.2 Text generation

Once obtained, the lists of facts were utilized to produce texts. From each list of facts, a text was generated paragraph by paragraph, each paragraph (except the first) created using knowledge of all preceding paragraphs, the target length, and the key fact it was required to include, as depicted in Figure 2b. Generation was performed using GPT-4o,^4^ selected for its ability to produce the exact number of requested sentences when that number was below 10. However, due to instability in generating longer paragraphs, the LLM was directed to create multiple smaller paragraphs (each with 5 sentences), which were subsequently combined. This approach, such as generating a 10-sentence paragraph by combining two 5-sentence paragraphs, yielded results comparable to directly producing a 10-sentence paragraph. A tolerance of one sentence between the target and actual length was permitted during length verification.

#### 2.2.3 Evaluation

##### 2.2.3.1 Text scoring

Summaries were assessed by examining the presence of facts from the original text generation, using GPT-4o.^5^ To verify whether these facts appeared in the summaries, the original list of facts was supplied to GPT-4o alongside each summary, with instructions to determine the presence or absence of each fact.

For each text, including the original generated text, the number of retrieved facts was calculated. Although text generation was nearly perfect, some facts occasionally failed to appear in the generated text. To account for this, only facts confirmed as present in the original text were considered when scoring the summaries. For a given summary, the score was calculated using the following formula, where *N*_*s*_ represents the number of facts retrieved in the summary and *N*_*o*_ denotes the number of facts retrieved in the original text:


(1) $$\begin{array}{l} score=\frac{N_{s}}{N_{o}} \end{array}$$

##### 2.2.3.2 Score analysis

Once scores were obtained, an analysis was conducted for each parameter set. The objective was to examine the score as a function of the summarization method applied and the SLM utilized, while accounting for the intrinsic variability of each text. For this purpose, a linear mixed-effects model (Pinheiro and Bates, 2000) with a random effect for text was fitted. A Sidak correction was employed to adjust the degrees of freedom. With *Id* representing the text identifier, the model formula is expressed as follows:


(2) $$\begin{array}{l} Score\sim SLM+Method+SLM:Method+\left(1|Id\right)+\epsilon \end{array}$$

where *SLM* denotes the summarization model, with five possible options (Gemma2:2b, Llama3.2, Openhermes:v2.5, Llama3.1 and Gemma2:9b) and Method represents the summarization approach (Stuff or Map).

After fitting the model, statistical analysis was performed to assess the significance of both the interaction between *SLM* and *Method* and the main effects. *Post-hoc* tests were conducted for mean comparisons, and the magnitude of differences was quantified using Cohen's D. Lettering has been attributed to each mean value, representing the differences between them. Specifically, two values with the same lettering are not significantly different.

##### 2.2.3.3 Error analysis

After scores were analyzed at the text level, additional investigations were conducted to identify which facts were absent from the summaries. One hypothesis posited that the Map method mitigates the “Lost in the Middle” effect, enabling better inclusion of facts located in the middle of the original text. By design, the position of each fact within the original text is theoretically known, since each fact corresponds to a paragraph. However, the study design precluded analysis of exact positions (each position per document was assessed only once), so facts were grouped into three categories. With the number of paragraphs being a multiple of 3, texts were organized into sets of two, three, or four contiguous paragraphs for texts with 6, 9, or 12 paragraphs, respectively. For texts with 6 paragraphs, the first two were designated as the beginning, the next two as the middle, and the final two as the end; for 9-paragraph texts, the first three, middle three, and last three were used, and for 12-paragraph texts, the first four, middle four, and last four were applied accordingly.

Consistent with the previous section, the probability of retrieving facts for a given small language model (SLM) across these cases was compared using a logistic mixed-effects model, with a Sidak correction applied to adjust the degrees of freedom. The model formula, where Pos represents the position (Beginning, Middle, or End) and Retrieved denotes the probability of fact retrieval, is given as follows:


(3) $$\begin{array}{l} Retrieved\sim Method+Pos+Method:Pos+\left(1|Id\right)+\epsilon \end{array}$$

Once the model fitted, statistical analysis was applied to explore the significance of the interaction between *Pos* and *Method* and the principal effects. *Post-hoc* tests for means comparison were applied.

##### 2.2.3.4 Comparison with LLM

To compare the performance of SLMs using the Map method with that of a LLM, the Stuff method was applied using the LLM GPT-4o, with an estimated parameter count exceeding one trillion (Shahriar et al., 2024), known for achieving state-of-the-art performance on many benchmarks. For each combination of paragraph number and paragraph size, texts were summarized using GPT-4o with the Stuff method exclusively. The objective was to determine whether SLMs employing the Map method could approach the performance levels of a LLM utilizing the Stuff method conventionally.

Scores were assigned to each summary generated by GPT-4o, consistent with the methodology of prior sections. A linear mixed-effects model, incorporating a random effect for text, was fitted to analyze the results. A Sidak correction was employed to adjust the degrees of freedom. With *Id* representing the text identifier and *LLM* denoting the model (SLMs with the MAP method, except for GPT-4o with the Stuff method), the model formula is presented as follows:


(4) $$\begin{array}{l} scores\sim LLM+\left(1|Id\right)+\epsilon \end{array}$$

After fitting the model, statistical analysis was conducted to assess the significance of the model effects, accounting for the respective methods. *Post-hoc* tests were performed for mean comparisons.

### 2.3 Practical study

To validate the results observed in the simulation study, a practical study was conducted. The purpose of this study was to summarize scientific papers and compare the resulting summaries with the authors' abstracts. A new dataset, tailored for this task, was compiled using freely accessible articles segmented into sections.

#### 2.3.1 Data

To validate our simulation results, we compiled a corpus for a practical study using scientific articles from six open-source Frontiers journals (Frontiers Media, 2024a,b,c,d,e,f), all classified as original research. Due to SLMs' limited context management, only papers with fewer than 6,000 tokens were retained to ensure the Stuff method could process inputs without truncation. This token limit was determined based on the smallest SLM context length (Gemma2 with only 8,000 tokens, input and output included) with a 2,000-token margin allocated for response generation. Retracted articles were excluded from the dataset.

Only the main body of each article, from introduction to conclusion, was included, omitting sections like Acknowledgments or Conflicts of Interest. The abstract was treated as the gold standard for summarization and thus excluded from the summarization process. For the Map method, texts were simply divided into sections. The number of collected and retained articles is presented in Table 2.

**Table 2:** Articles by scope and number of articles kept; from a total of 5,331 articles, only 587 met the criteria and were kept in the final dataset.

**Scope of journal**	**Number of articles scrapped**	**Number of articles kept**
Artificial intelligence	670	83
Astronomy	208	43
Biotechnology	1,024	128
Earth Science	272	42
Education	2,453	160
Neuroscience	704	131

The statistical description of the corpus, including the number of sections, tokens, mean number of sentences per section, and standard deviation of sentences per section, is presented in Table 3.

**Table 3:** Statistics of articles kept in corpus.

**Statistic**	**Number of sections**	**Number of tokens**	**Mean number of sentences by sections**	**Std number of sentences by section**
Mean	5.51	4,414.54	27.8	16.92
Std	1.62	626.49	7.49	6.21
Min	3	2,141	8.50	2.60
Max	13	5,468	59.75	51.28

#### 2.3.2 Evaluation

As in the simulated study, both the Stuff and the Map methods were applied to each article to produce a summary. The objective was to identify which method yielded a summary closest to the abstract, considered as the reference.

##### 2.3.2.1 Summaries evaluation

The use of SLM produces summaries with synonyms and paraphrases of the original text. To prioritize semantic content over vocabulary [as would the ROUGE metric (Lin, 2004), whose limitations are presented in Sai et al. (2022) and Zhang et al. (2024)], evaluation was performed by computing the cosine similarity between the abstracts and the summaries (Wang and Dong, 2020).

Both summaries (generated by the Stuff and Map methods) and the corresponding abstracts were first vectorized using a Longformer (Beltagy et al., 2020) to convert each text into a 1,024-dimensions vector. A Longformer was used due to the text lengths exceeding the context limits of standard Transformers. The specific Longformer employed was a fine-tuned version of the original longformer-large-4,096, further pretrained on the S2ORC corpus (Lo et al., 2019), which comprises academic papers across various domains, rendering it well-suited for this task. After vectorization, the cosine similarity between each summary and its corresponding abstract was calculated. The cosine similarity between two vectors *u* and *v* is given by:


(5) $$similarity(u,v)=\;\frac{u\cdot v}{{\left|u\right|}_{2}{\left|v\right|}_{2}}$$

However, cosine similarity has some limitations. The embedding was obtained by computing the mean of token embeddings, resulting in a mean representation of the text. Consequently, some of the granularity of the text is lost. Additionaly, cosine similarity is unaffected by text length, meaning a long, detailed summary and a short, dense summary could could yield comparable scores. Furthermore, cosine similarity is dimensional sensitive, tending to concentrate around mean values, which can make it challenging to distinguish between low- and high-quality summaries.

To address these limitations, the BERTScore (Zhang et al., 2019) was used to compare the generated summaries and their corresponding abstracts. The BERTScore embeds each token in the summaries and abstracts individually. Each token is then matched to the most similar token between the summary and abstract to compute a similarity score. For a summary $\hat{x}$ and its corresponding abstract *x*, BERTScore's recall, precision and F1-score are defined as in Zhang et al. (2019):


(6) $$\begin{array}{l} R_{BERT}=\frac{1}{\left|x\right|}\underset{x_{i}\in x}{\sum }\underset{{\hat{x}}_{j}\in \hat{x}}{\max}x_{i}^{⊤}{\hat{x}}_{j} \end{array}$$

(7) $$\begin{array}{l} P_{BERT}=\frac{1}{\left|\hat{x}\right|}\underset{{\hat{x}}_{j}\in \hat{x}}{\sum }\underset{x_{i}\in x}{\max}x_{i}^{⊤}{\hat{x}}_{j} \end{array}$$

(8) $$\begin{array}{l} F_{BERT}=2\frac{P_{BERT}\cdot R_{BERT}}{P_{BERT}+R_{BERT}} \end{array}$$

##### 2.3.2.2 Scores analysis

For each SLM and summarization method, the mean cosine similarity, BERTScore precision, recall and F1-score across the corpus were calculated, accompanied by their corresponding 95% confidence interval, as outlined in Equation 9, where *X* represents the vector of values; $\bar{X}$ denotes the mean, *N* indicates the number of values, and *std*(*X*) signifies the standard deviation. Additionally, to assess the potential advantage of the Map method over the Stuff method, paired *t*-test were conducted, supplemented by an estimation of effect size using Cohen's d.


(9) $$\begin{array}{l} IC=\left[\bar{X}-1.96\times \frac{std\left(X\right)}{\sqrt{N}};\bar{X}+1.96\times \frac{std\left(X\right)}{\sqrt{N}}\right] \end{array}$$

## 3 Results

### 3.1 Simulation results

#### 3.1.1 Comparison between map and stuff according to several SLMs

**Figure 3:**
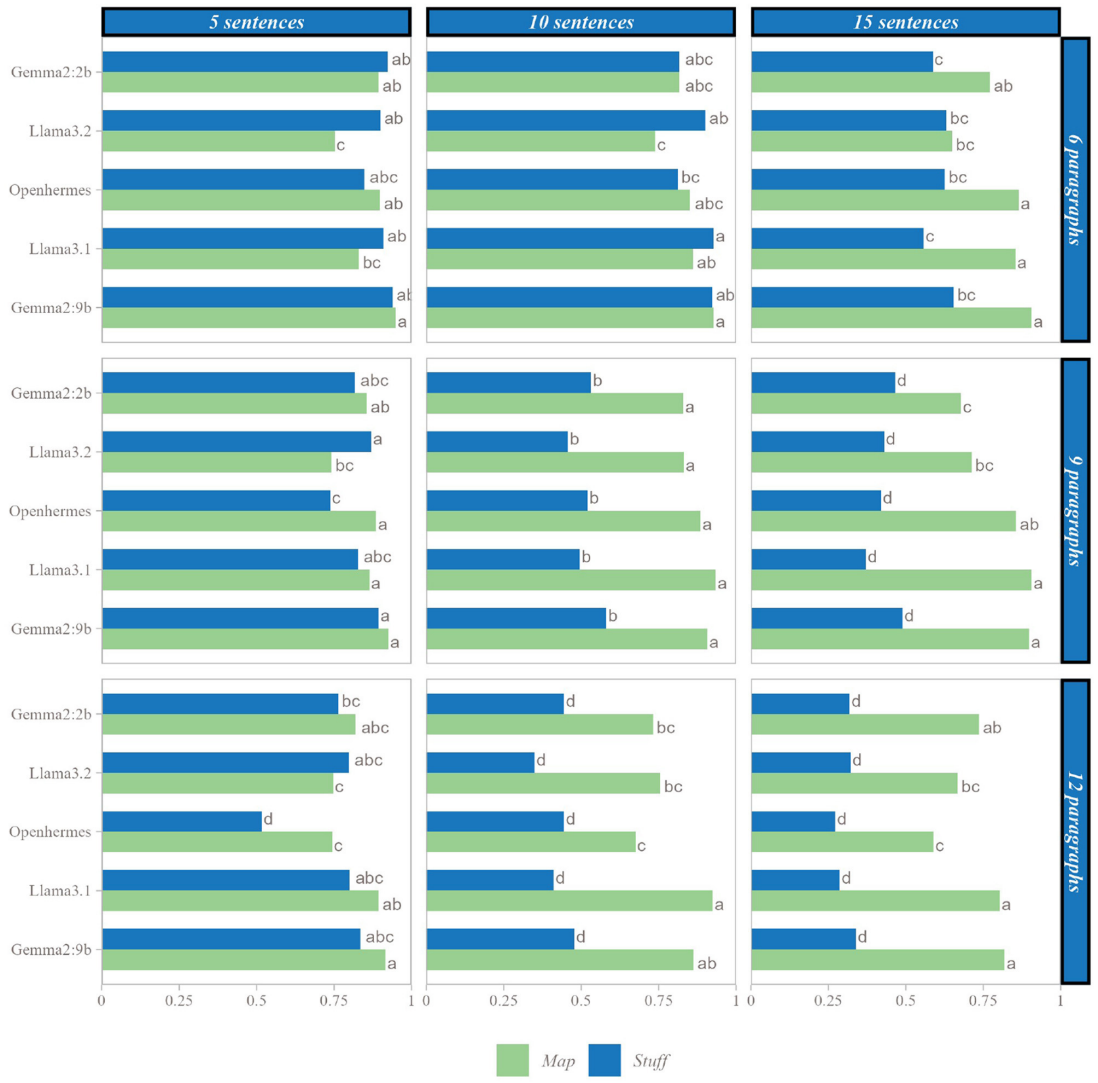
Comparison of SLM performances on each case, mean scores with the same letter are not statistically different, within the number of paragraphs and sentences per paragraph considered.

The Map and Stuff methods were evaluated across six distinct SLMs using nine parameter sets. Figure 3 presents the mean scores achieved by each SLM with each method across these different parameter sets. When comparing methods within each SLM, no significant difference was observed between the two methods for shorter texts (under 90 sentences), except in specific cases: Llama3.2 showed significantly higher performance with the Stuff method for texts with 6 paragraphs of 5 sentences, 6 paragraphs of 10 sentences, and 9 paragraphs of 5 sentences. Conversely, Openhermes exhibited significantly better performance with the Map method for texts with 9 paragraphs of 5 sentences and 12 paragraphs of 5 sentences. However, as text length increased, a clear divergence emerged between the methods, with the Map method demonstrating superior performance across all SLMs.

When comparing the SLMs, for texts exceeding 9 paragraphs with more than 10 sentences, a smaller model such as Gemma2:2b using the Map method outperformed a larger model like Gemma2:9b using the Stuff method. For instance, in the extreme case of 12 paragraphs with 15 sentences, mean scores were 0.736 for Gemma2:2b with the Map method and 0.339 for Gemma2:9b with the Stuff method.

**Figure 4:**
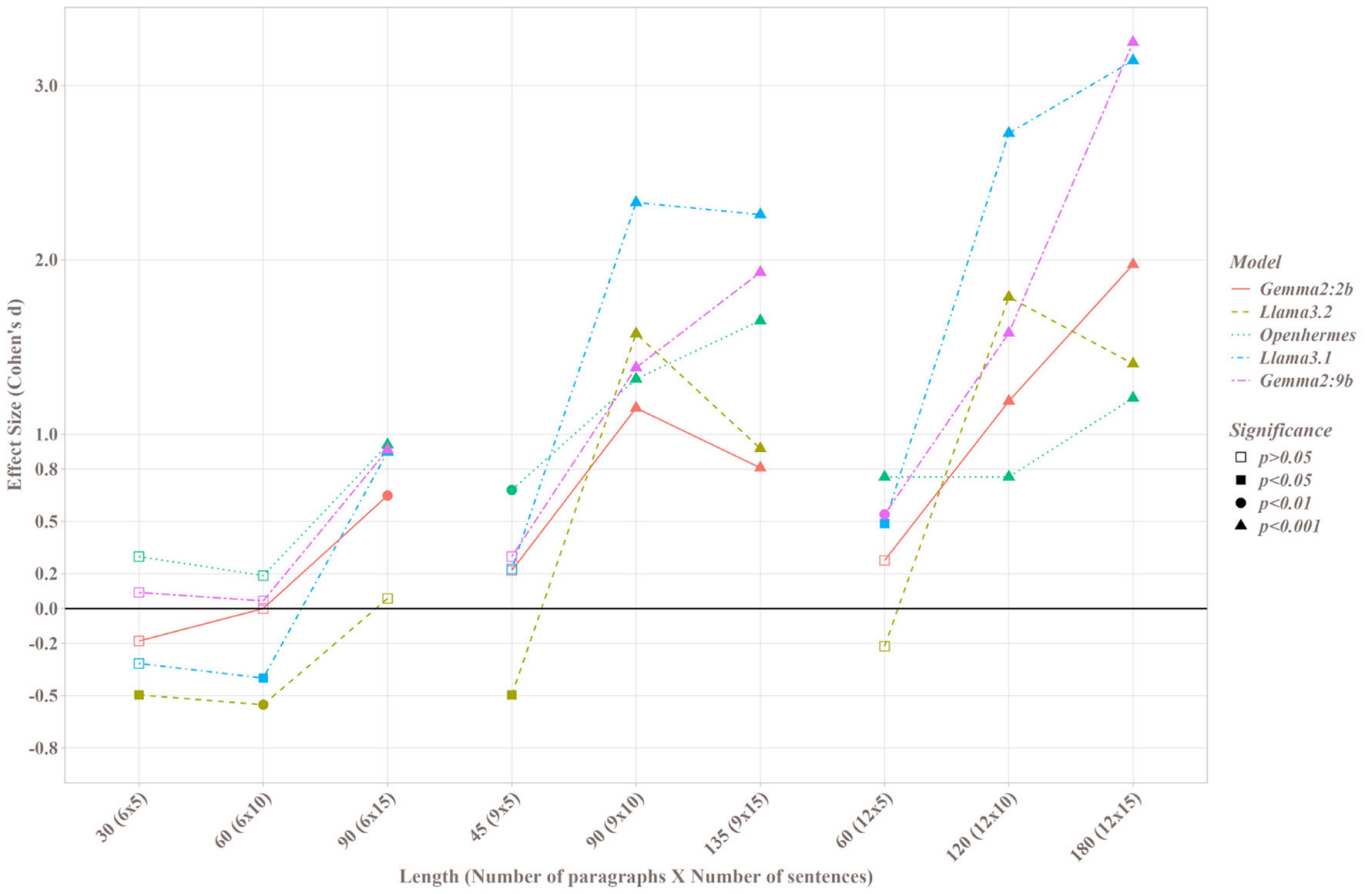
Cohen's d value and paired t-test's significance between the Map and Stuff methods for each SLM on each set of parameters; p corresponding to the p-value of the paired *t*-test.

Figure 4 illustrates the significance levels and effect sizes from paired t-tests comparing the Map and Stuff methods. Across all SMLs and parameter sets, the Map method outperformed the Stuff method when differences were statistically significant, with the sole exception of the Llama3.2 model. For Llama3.2, three parameter sets (6 paragraphs of 5 sentences, 6 paragraphs of 10 sentences, and 9 paragraphs of 5 sentences) yielded negative Cohen's d values, indicating superior performance of the Stuff method in these cases. For nearly all models, increases in the number of paragraphs or paragraph length enhanced the advantage of the Map method over the Stuff method. However, with Llama3.2, the benefit of the Map method appeared diminished for paragraphs of 15 sentences compared to those of 10 sentences. When considering the total sentence count within texts, the Map method exhibited significant superiority over the Stuff method for counts exceeding 90 sentences, often with very large effect sizes (greater than 1 for all SLMs). The largest effect size recorded was 3.249 for Gemma2:9b on the longest texts.

#### 3.1.2 Effect of position

**Figure 5:**
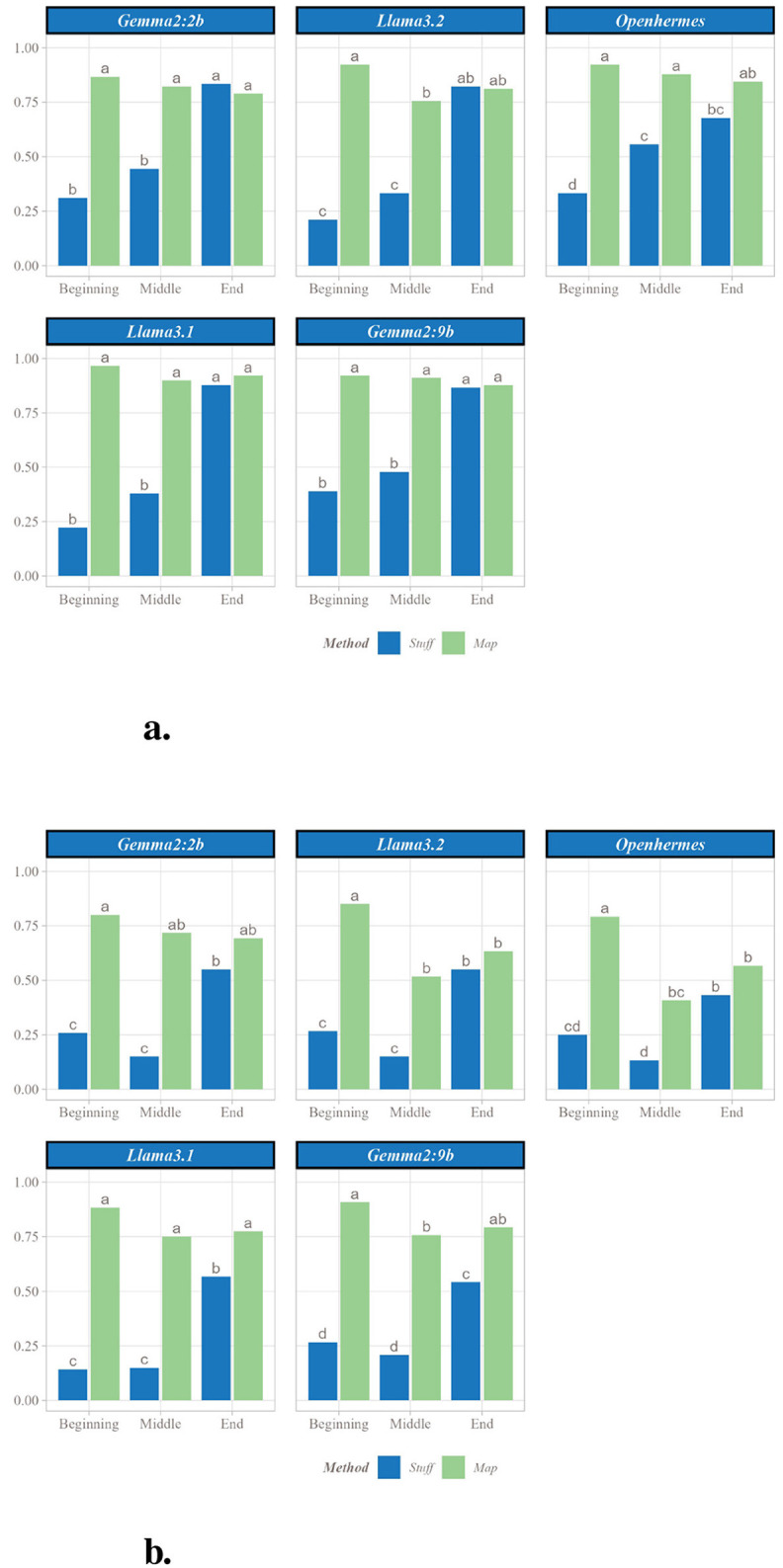
Analysis of proportion of fact retrieved by position in the original text. **(a)** Proportion of facts retrieved with each SLM by position in original text (for 9 facts and 10 sentences). **(b)** Proportion of facts retrieved with each SLM by position in original text (for 9 facts and 10 sentences). Letters represent significant differences: two groups presenting the same letter are not significant different.

Given the observed performance differences between the methods, additional analyses were conducted to investigate their origins. Two parameter sets were examined: the first, consisting of 9 paragraphs with 10 sentences (90 sentences total), represents the threshold where significant differences between the Stuff and Map methods emerge, as shown in Figure 5a; the second, comprising 12 paragraphs with 15 sentences (180 sentences total), corresponds to the cases exhibiting the largest effect sizes, as depicted in Figure 5b.

Both parameter sets revealed that, for nearly all SLMs, facts located at the beginning and middle of the text were less frequently retrieved with the Stuff method compared to those at the end. In contrast, the Map method consistently captured facts regardless of their position in the original text. Consequently, the Map method's advantage over the Stuff method was more pronounced for facts at the beginning and middle of the text than at the end. For instance, in the 90-sentence case (Figure 5a) with Gemma2:9b, the Stuff method retrieved 38.9% of facts from the beginning, 47.8% from the middle, and 86.7% from the end, whereas the Map method retrieved 92.2%, 91.1%, and 87.8%, respectively. Thus, the Map method recovered over twice as many facts from the beginning and middle compared to the Stuff method, while retrieval rates at the end remained comparable.

#### 3.1.3 Comparison with LLM

**Figure 6:**
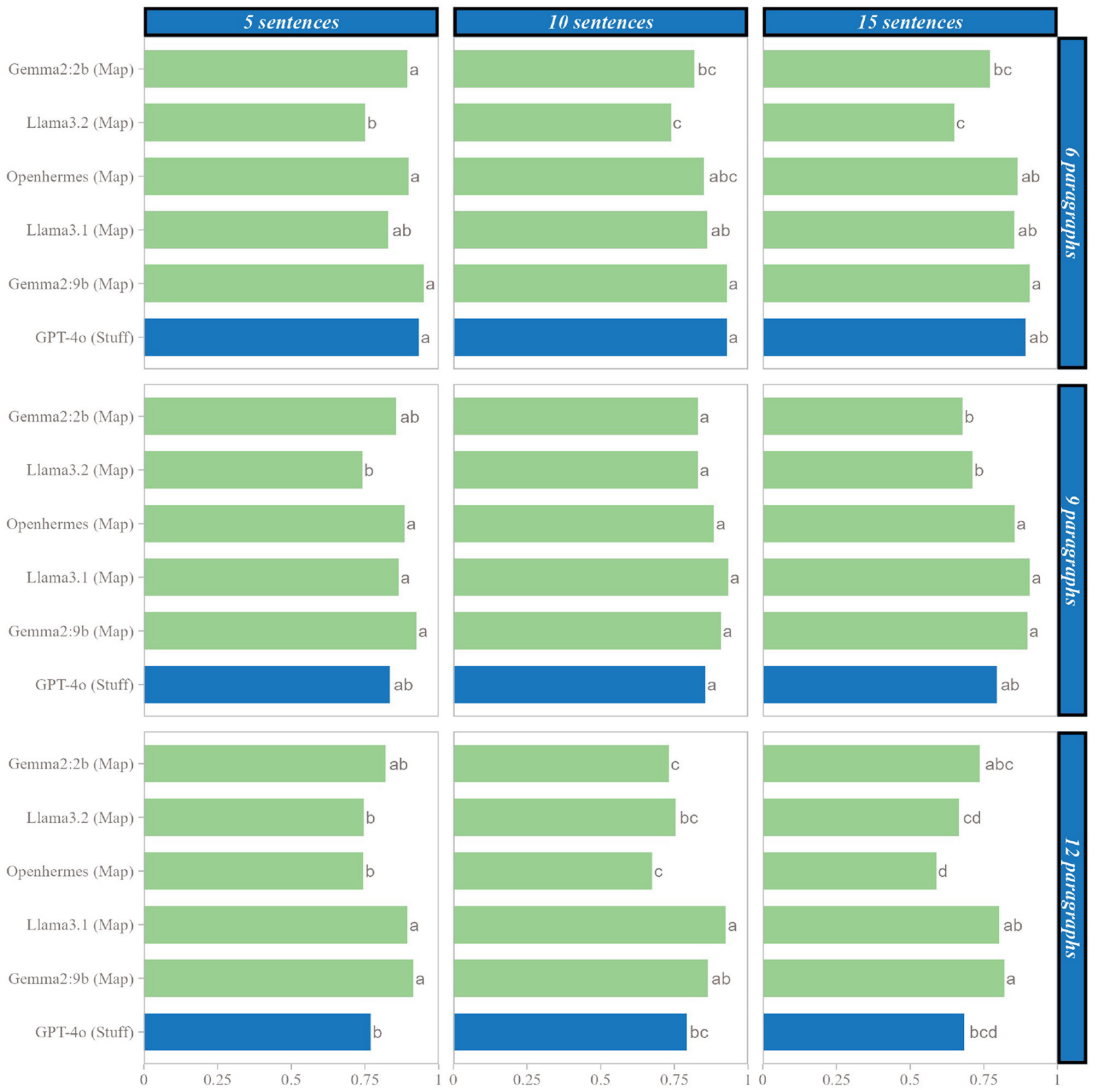
Comparison of SLMs with the Map method and LLMs with the Stuff method for each number of paragraph and each length of paragraph. Letters represent significant differences: two groups presenting the same letter are not significant different.

Figure 6 illustrates the performance of SLMs using the Map method compared to GPT-4o using the Stuff method. *Post-hoc* tests revealed that nearly all SLMs achieved performance levels with the Map method comparable to those of GPT-4o with the Stuff method. Exceptions included Llama3.2 with 6 paragraphs—regardless of sentence count - and Gemma2:2b with 6 paragraphs of 10 sentences, where performance was significantly lower than that of GPT-4o. Conversely, for texts with 12 paragraphs, Llama 3.1 and Gemma2:9b demonstrated significantly higher performance than GPT-4o, specifically with 5 and 10 sentences for Llama3.1 and 5 and 15 sentences for Gemma2:9b.

### 3.2 Practical results

In this practical study, the summarization performance of five SLMs using the Stuff and Map methods was assessed. The study utilized a corpus of 587 scientific articles to determine which method produced summaries most aligned with the authors' abstracts.

**Figure 7:**
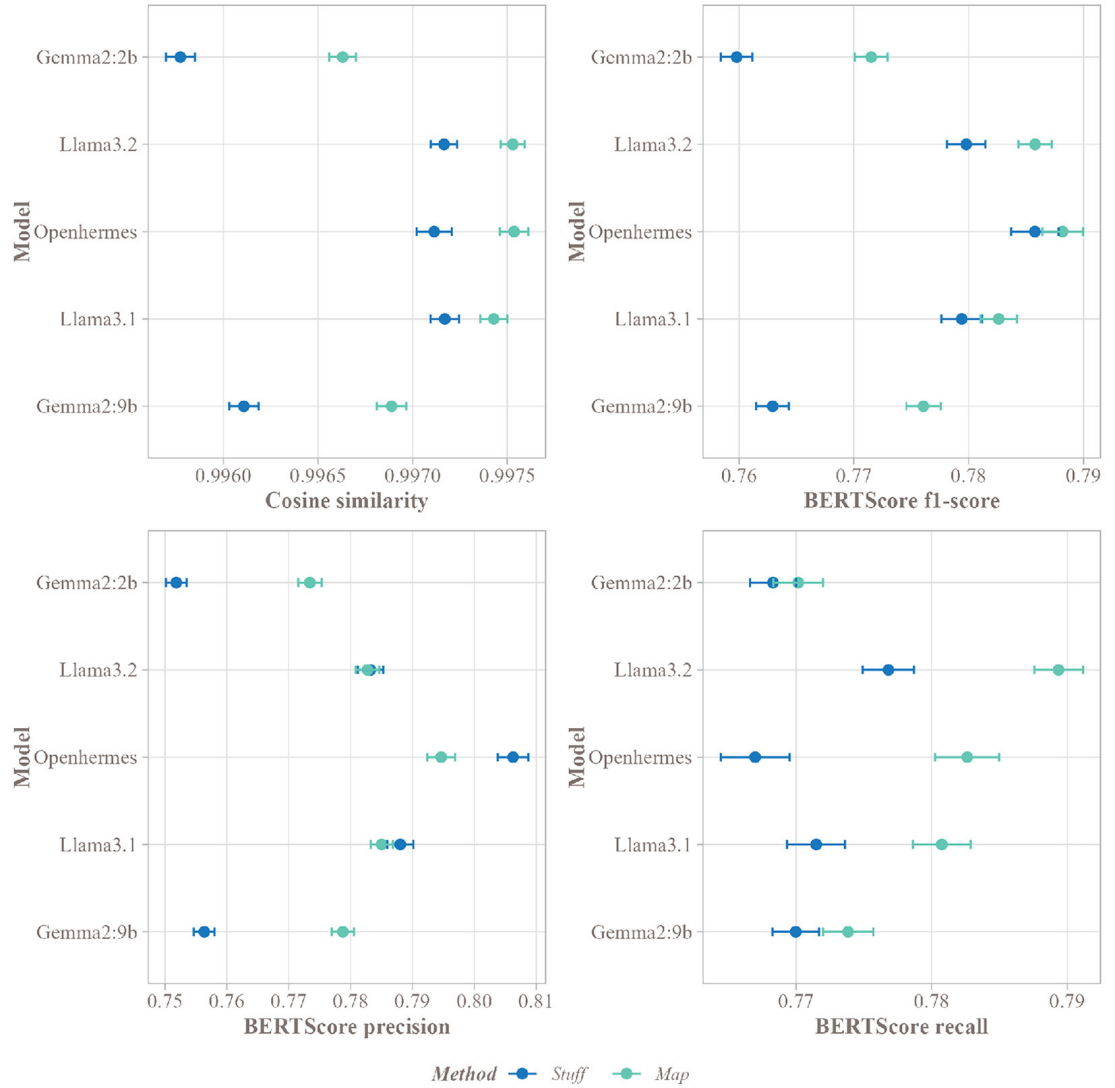
Mean of the different scores for the Stuff and Map methods for each SLM on the corpus of articles, with a 95% confidence interval.

**Figure 8:**
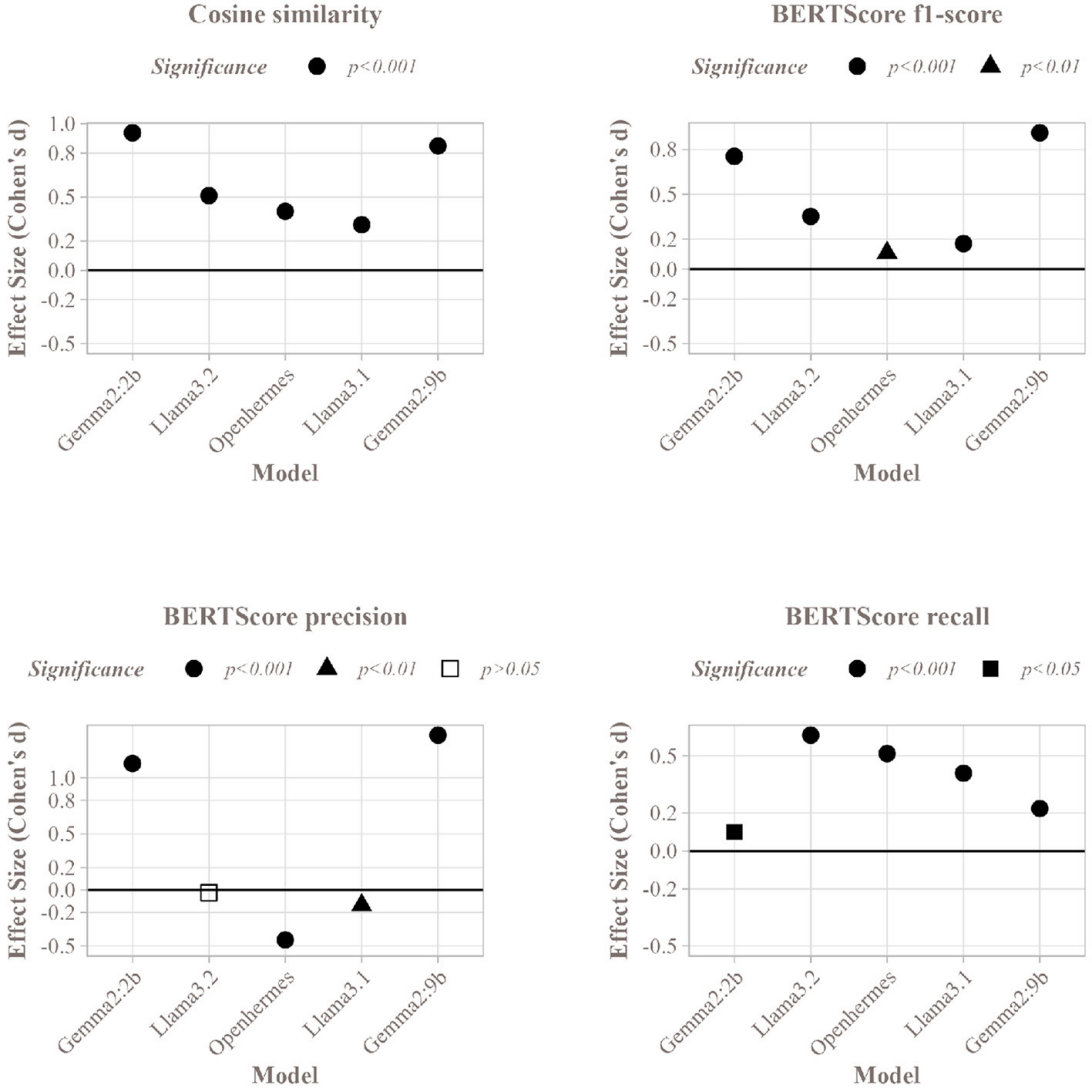
Cohen's d value and paired *t*-test significance of the Stuff and Map methods for each SLM on the corpus of articles, for the different metrics used.

Figure 7 displays the results of this comparison. Overall, high cosine similarity scores (above 0.995) were observed across all models, indicating strong correspondence between summaries and abstracts. However, the Map method significantly outperformed the Stuff method in matching with the authors' abstracts across all SLMs. Concerning BERTScore, precision was higher for Gemma2:2b and Gemma2:9b, with significant p-values below 0.001 (Figure 8), indicating that a larger proportion of summaries aligned with the abstract for these models. Openhermes showed no difference between the Stuff and Map methods, while Llama3.1 and Llama3.2 had better precision with the Stuff method. However, regarding recall, all models performed better with the Map method, indicating that a greater proportion of the abstract was captured by these summaries, resulting in less information loss during the summarization process. Finally, the F1-score was higher for all SLMs using the Map method, suggesting that summaries generated with this method were of higher quality.

Given the subtle differences in cosine similarity scores (sometimes as small as 0.001), effect sizes were computed to evaluate the significance and magnitude of these differences. Figure 8 presents the effect sizes for all five SLMs. The Gemma models exhibited strong positive effect sizes (above 0.8), highlighting the substantial benefit of the Map method over the Stuff method for these models. In contrast, the Llama models and Openhermes:v2.5 displayed weaker positive effects (above 0.2 for Llama3.1 and Openhermes:v2.5, and above 0.5 for Llama3.2), suggesting a less pronounced but still favorable advantage for the Map method. Nevertheless, despite the small differences in cosine similarity, the Map and Stuff methods produced summaries with very similar proximity to the abstract. Regarding BERTScore F1-score, Gemma2:2b and Gemma2:9b exhibited the highest effect sizes, consistent with cosine similarity results. Other models also showed positive effect sizes, though slightly weaker.

## 4 Discussions

In this paper, two questions regarding summarization with SLMs were explored. The first examined whether a summarization method based on text splitting (Map method) could outperform a conventional approach of providing the entire text to the SLM at once (Stuff method) for short texts. The second investigated whether the Map method could mitigate the “Lost in the Middle” effect. To address these questions, a simulation study and a practical application were conducted.

Simulated texts were generated by creating facts with GPT-3.5-turbo and producing text from these using GPT-4o. These two models were selected for their efficiency in their respective tasks at the time of the study and performed reliably with minimal divergence. Different SLMs were used for summarization to avoid overlap. Only the comparison with an LLM used a GPT model for summarization, but the results showed lower scores, suggesting no advantage. The evaluation also employed GPT-4o, which could introduce bias, but it was the most effective model for this task with few errors. Futhermore, performance was not assessed individually but by comparing the two methods, which were subject to the same bias.

Both the simulation and practical studies demonstrated that the Map method produced summaries at least as effective as those generated by the Stuff method. Moreover, the simulation study revealed that the advantage of the Map method over the Stuff method increased with the length of the original text. Regarding the “Lost in the Middle” effect (Liu et al., 2024), positional analysis in the simulation study indicated a more pronounced “Lost in the Beginning” effect, as the Stuff method retrieved fewer facts from the first two-thirds of the original text. In contrast, the Map method effectively addressed this issue, retrieving facts nearly uniformly regardless of their position in the text. When comparing SLM performance with the Map method to GPT-4o with the Stuff method, the simulation showed that, for the task and evaluation process considered, SLMs bridged the performance gap with the well-established LLM. Notably, in the most complex cases, the two largest SLMs using the Map method achieved significantly better results than GPT-4o using the Stuff method.

The practical application involved use of scientific articles, which were longer than the studied text within the theoretical part, resulting in non-uniform sentence counts for the Map method's segments. However, the same conclusions were observed: the Map method outperformed the Stuff method, consistent with prior literature showing its effectiveness for long document summarization tasks (Wang et al., 2024; Moro and Ragazzi, 2022).

The evaluation process for the theoretical component relied on the initial list of facts, yet additional significant facts may have emerged during text generation. Such facts could be deemed more critical for summaries and prioritized over the original facts, particularly when generating long paragraphs. To enforce fixed-length paragraphs, the SLM was tasked with producing multiple short paragraphs that were subsequently concatenated, potentially increasing the likelihood of generating unaccounted yet important facts not reflected in the score. Nonetheless, this generation and evaluation approach eliminated the need for labor-intensive and time-consuming human evaluation. For the practical component, article abstracts were treated as the gold standard for summarization, though this choice has limitations, as abstracts may not encompass all key facts and are often highly structured.

The simulation established in this study offers a tool for comparing the summarization capabilities of two prompts on short texts. The comparison focuses exclusively on the facts within the texts, overlooking other essential aspects of a quality summary, such as the length, clarity, or linguistic quality, yet it avoids the need for human evaluation. Additionally, a practical dataset of scientific articles compatible with the context size of SLMs was developed. This dataset enables prompt or model comparisons tailored to specific requirements.

Although the Map method outperformed the Stuff method, it incurs higher costs due to increased SLM calls. However, it permits the use of smaller SLMs, which reduces per-call computational costs and energy consumption compared to LLMs, and lessens infrastructure demands, as larger SLMs typically require more robust GPU units. A comprehensive cost analysis was not conducted in this study but merits investigation in future research.

While the Map method's performance is unaffected by split size for long documents (Chang et al., 2023), the impact of the number of splits on short documents remains unexplored and could be a focus of future studies. Alternative splitting methods for long texts, such as incrementally refining a summary split-by-split, may also warrant examination. Nonetheless, splitting input text into smaller segments poses challenges, and the size of each split could influence performance-an aspect not addressed here but suitable for future exploration.

## 5 Conclusion

This study evaluated a summarization method (Map method), typically applied to long texts with SLMs, to address the “Lost in the Middle” effect. Additionally, a novel generation and evaluation process was introduced, enabling comparison of summarization methods without human evaluation. The hypothesis posited that the Map method would capture more information from the middle of texts compared to the Stuff method, which involves providing the entire text to the SLM. The simulation study revealed that the Stuff method struggled to include facts from the beginning and middle of texts. In contrast, the Map method, as anticipated, effectively incorporated facts regardless of their position, performing at least as well as the Stuff method and surpassing it in the most complex cases. This finding was corroborated in a practical study involving the summarization of scientific papers. Unexpectedly, the simulation study demonstrated that larger SLMs using the Map method outperformed a state-of-the-art LLM using the Stuff method in these challenging scenarios. For practitioners, these results suggest that the Map method generally offers greater effectiveness across most SLMs.

## Data Availability

The raw data supporting the conclusions of this article will be made available by the authors, without undue reservation.

## Footnotes

^1^Prompt for Stuff method is available in Supplementary material.

^2^Prompts for Map methods are available in Supplementary material.

^3^The prompt used to generate facts (excluding the few-shot examples) is provided in Supplementary material.

^4^Prompts for text generation are available in Supplementary material.

^5^The prompt employed for this evaluation is available in Supplementary material, such as a repeatability analysis of the evaluation method.
