# Supplementary material for: Divide and summarize: improve SLM text summarization
Source: Front Artif Intell. 2025 Aug 1;8:1604034. doi: 10.3389/frai.2025.1604034 (PMC12354567; doi:10.3389/frai.2025.1604034)
Supplement: Supplementary file 1 [file Data_Sheet_1.pdf]

# Supplementary Material

## 1 LIST OF PROMPTS

**Table S1.** Lists of prompts used in this study

| Use case | Prompt | Explanation |
|---|---|---|
| Stuff summarization | Write a summary of the following text:<br>'TEXT'<br>SUMMARY: | TEXT is the text to summarize |
| Map partial summarization | Write a summary of the following text:<br>'TEXT'<br>SUMMARY: | TEXT is the partial text to summarize |
| Map regroupment summarization | The following is a set of summaries of the different parts of a text:<br>'SUMMARIES'<br>Take these and use these to write a summary of the original text.<br>SUMMARY: | SUMMARIES is the list of partial summaries to summarize |
| List of fact generation | Provide $N_l$ lists of $N_f$ short facts that are the main facts of $N_f$ independent fictional stories.<br>Facts on the same list must be related.<br>The facts must be different and not related from one list to another one.<br>Facts must be concise and not too detailed.<br>Only provide the output. | $N_l$ and $N_f$ are respectively the number of list and the number of facts by list |

| Use case | Prompt | Explanation |
|---|---|---|
| Text generation from list of facts (first paragraph) | Topic :<br>—<br>'FACT'<br>—<br><br>'N' paragraphs of 5 sentences: | FACT is the fact used as main topic and N is used to control the number of sentences within the paragraph (1 for a 5 sentences, 2 for 10 sentences,...) Paragraphs are then concatenated |
| Text generation from list of facts (other than first paragraph) | Beginning of story:<br>—<br>'BEGIN'<br>—<br><br>Topic :<br>—<br>'FACT'<br>—<br><br>'N' paragraphs of 5 sentences: | FACT is the fact used as main topic, BEGIN is the previously generated paragraphs and N is used to control the number of sentences within the paragraph (1 for a 5 sentences, 2 for 10 sentences,...) Paragraphs are then concatenated |
| Fact retrieval | You are an assistant verifying if facts are present in texts.<br>Given a text and a list of facts, you must say if the facts are present in the text.<br>Just answer with a list of boolean 'True' or 'False' for each fact.<br>Be sure to give an answer to each fact.<br>The number of facts to evaluate is provided by the user.<br>Let's think step by step, one fact at a time.<br>The answer must only be a list like that: [bool, bool, bool] (for 3 facts in this example).<br><br>Number of facts : 'N'<br>—<br>Facts : 'FACTS'<br>—<br>Text : 'TEXT' | N is the number of facts to looking for, FACTS is the list of facts to looking for and TEXT is the text within looking for facts |

## 2 REPEATABILITY ANALYSIS OF EVALUATION METHOD

Repeatability analyzes for the evaluation process used in simulated part were conducted and presented in this appendix. The evaluation consisted in providing GPT-4o with a list of facts, the number of facts in the list and a text, then instructing the model to determine for each fact whether it was present in the text . To assess the consistency of this method, 2700 facts were evaluated across 1,440 texts, with the evaluation process repeated three times for each fact in each document.

To verify the model's consistency in these assessments, two logistic mixed-effects models were constructed to predict fact retrieval. The first model excluded the evaluation's number, while the second included it. The equations for these models are as follows:

$$Retrivied \sim 1 + (1|Id_{fact}) + (1|Id_{doc}) \tag{S1}$$

$$Retrivied \sim N_{eval} + (1|Id_{fact}) + (1|Id_{doc}) \tag{S2}$$

Here, $Id_{fact}$, $Id_{doc}$ and $N_{eval}$ represent the fact identifier, document identifier, and evaluation's number, respectively.

To confirm that $N_{eval}$ had no influence, two tests were performed. First, the second model's parameters related to the evaluation's number were examined for significance. Second, a likelihood ratio test was conducted to compare the two models and determine whether including the evaluation's number significantly improved the fit.

## 2.1 Parameters analysis

The coefficients from the model incorporating the evaluation number were analyzed. With three evaluations and the first serving as the reference, two coefficients were estimated: one for $N_{eval} = 2$ and one for $N_{eval} = 3$. The estimated values were 2.2997e-2 and -7.038e-3 with p-values of 0.614 and 0.906, respectively. Given these p-values, neither coefficient was significantly different from zero, indicating that the probability of fact retrieval in a document remains unaffected by the evaluation number.

## 2.2 Likelihood Ratio Test (LRT)

Likelihood Ratio Test (LRT) was employed to compare the nested models and assess whether the more complex model significantly enhanced data fit. Under this test, the null hypothesis posits that the simpler model sufficiently explains the data. The test statistic is calculated as:

$$D = -2 \times (l_0 - l_1) \tag{S3}$$

where $D$ is the test statistic, and $l_0$ and $l_1$ are the log-likelihoods of the simpler and more complex models, respectively. Under the null hypothesis, D follow a $\chi^2$ distribution with degrees of freedom $(df)$ equal to the difference in the number of parameters between the models (2 in this case).

The LRT statistic obtained was $D = 0.4382$, with a corresponding p-value of 0.8032 at a threshold of $\alpha = 0.05$. As the null hypothesis was not rejected, the simpler model adequately fits the data.

## 2.3 Conclusion

The number of evaluations does not affect the prediction of fact retrieval. Consequently, GPT-4o's output remains consistent across repeated evaluations, confirming the repeatability of the evaluation method.
